# From flat bones to functional healing: redefining rodent calvarial defect models for translational bone regeneration research

**DOI:** 10.1097/MS9.0000000000003528

**Published:** 2025-06-27

**Authors:** S. Amitha Banu, K. M. Manjusha, Merlin Mamachan, Khan Sharun

**Affiliations:** a Division of Surgery, ICAR-Indian Veterinary Research Institute, Izatnagar, Bareilly, Uttar Pradesh, India; b Graduate Institute of Medicine, Yuan Ze University, Taoyuan, Taiwan

**Keywords:** bone healing, bone tissue engineering, calvarial defect, tissue engineering, regenerative medicine

## Abstract

Preclinical models are essential for evaluating bone tissue engineering strategies, but their translational relevance is frequently debated. Rodent calvarial defect models (RCDs) are widely used due to their reproducibility, cost-effectiveness, and compatibility with genetic manipulation. Despite these advantages, their translational relevance remains controversial due to key anatomical and physiological differences from human bones. RCDs heal exclusively via intramembranous ossification and lack biomechanical loading, in contrast to the endochondral ossification and dynamic stress conditions characteristic of long bone healing in humans. Additionally, inconsistencies in defining critical-size defects (CSDs), variation in defect placement relative to cranial sutures, and the influence of age-related skeletal changes hinder cross-study comparisons and clinical extrapolation. These limitations underscore the need for standardized defect parameters, age-matched models, and advanced scaffolds with tunable degradation rates aligned with bone regeneration timelines. Therefore, adopting standardized protocols, integrating advanced biomaterials, and employing clinically relevant testing environments can substantially enhance the predictive power and translational relevance of calvarial defect models. While RCDs serve as a valuable platform for early-stage screening and mechanistic insights, strategic refinements in model design, paired with complementary validation in higher order species, are essential for bridging the gap between preclinical research and clinical application in bone tissue engineering.

## Introduction

**Figure 1.**
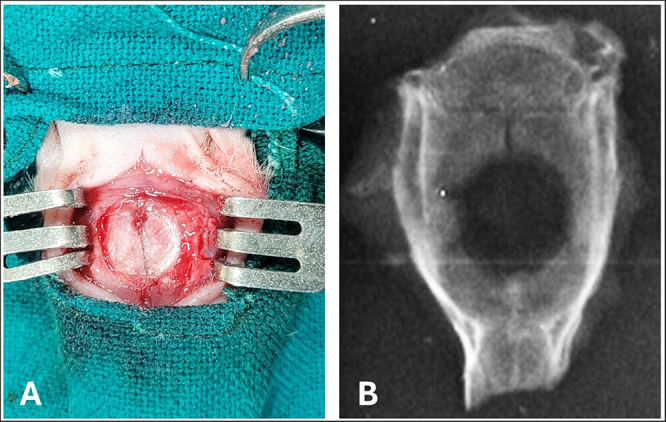
Critical-sized calvarial defect in a rat model. (A) Intraoperative view showing the surgical creation of a standardized full-thickness calvarial defect in a rat. (B) Radiograph illustrating the circular bone defect at day 45.

Bone tissue exhibits inherent regenerative capacity, but unfavorable microenvironments, suboptimal surgical techniques, biomechanical instability, or systemic conditions disrupting physiological equilibrium can impair healing. This necessitates biomaterial-based interventions for large defects exceeding natural repair capabilities^[1]^. It is well established that effective preclinical models for bone tissue engineering must replicate the clinical environment, enable quantitative assessment of tissue regeneration, and predict biological performance differences between strategies to facilitate clinical translation^[2]^. *In vitro* assays are invaluable for elucidating fundamental biological mechanisms and screening the biological activity, toxicity, and immunogenicity of compounds, but they cannot fully replicate the complexity of the *in vivo* environment or reliably predict clinical outcomes^[3,4]^. Animal models, especially calvarial defect models (CDM) (Fig. 1), offer a practical *in vivo* system for studying bone healing and reconstruction due to their minimal load-bearing nature of the skull, making it less affected by external forces than long bones^[5]^. The effectiveness of reconstructive interventions can be evaluated using radiological techniques like micro-CT, as well as histological and high-resolution cellular imaging modalities^[6-8]^. Rodent calvarial defect (RCD) models are widely used to study bone regeneration and biomaterial performance due to their reproducibility, cost-effectiveness, and compatibility with transgenic studies^[6]^. However, there is ongoing debate regarding their translational relevance due to factors such as variability in defect size, species differences, and the unique intramembranous healing pattern of calvarial bone^[9-11]^.

**HIGHLIGHTS**

- Rodent calvarial defect models (RCDs) are widely used for evaluating bone regeneration strategies due to their reproducibility and cost-effectiveness.
- The translational relevance of RCDs is limited by species-specific differences in healing, bone architecture, and mechanical loading.
- Intramembranous ossification in RCDs fails to replicate the endochondral healing mechanisms of clinically relevant long bone defects.
- Standardizing defect size, anatomical location, and animal age can improve cross-study reliability and model predictability.
- Translational testing in large animal models and engineered RCDs that promote endochondral ossification can bridge the gap to clinical application.

## Rodent calvarial defect model

A major point of debate in the use of CDMs for translational research is the choice of species and defect size. While large animals such as pigs and non-human primates more accurately replicate human cranial bone structure, healing dynamics, and clinical conditions, the majority of studies employ rodent models due to their convenience and cost-effectiveness^[10,12]^. However, rodents present several limitations, including faster bone healing rates, ongoing cranial growth, differences in bone microarchitecture and biomechanics, as well as restricted capacity for long-term studies, all of which reduce their ability to replicate human skeletal reconstruction^[1]^. A comprehensive analysis of postnatal craniofacial skeletal development in female C57BL/6NCrl mice revealed that the most rapid craniofacial growth occurs during the first three weeks after birth, with slower but continued growth and remodeling in specific bones up to 13 months of age^[13]^. Rodent bones typically have higher bone volume relative to tissue volume, more numerous but thinner trabeculae, and different patterns of bone connectivity compared to humans^[14,15]^. Additionally, the mineral density and mechanical strength of rodent bones differ, with rat bone being less dense and exhibiting distinct fracture stress values compared to human bone^[15]^. The shorter lifespan limits the duration over which bone healing and remodeling can be observed, whereas biological constraints prevent repeated biopsies or the collection of adequate blood samples. Additionally, their small body size restricts the creation of large defects and limits the amount of tissue available for evaluation, making it challenging to simulate the scale and complexity of human clinical scenarios^[1,16]^.

A key issue is the variability in defining critical size defects (CSDs) across studies. For instance, while 5 mm defects are considered critical in young rats, aged rats may require smaller defects (4 mm) to achieve non-healing status^[1,10,17]^. Similarly, juvenile mice demonstrate spontaneous healing of 3–5 mm defects, whereas adults fail to heal even smaller defects. This inconsistency stems from differing experimental timelines, species-specific healing capacities, and age-related biological changes, complicating cross-study comparisons and undermining translational predictability^[17]^. Another limitation is the absence of mechanical loading in calvarial models. Unlike load-bearing human bones (e.g., femur), the rodent calvarium experiences minimal biomechanical stress, making it unsuitable for evaluating biomaterial durability or functional integration under physiological forces^[1]^. While this simplifies surgical stabilization, it fails to replicate the mechanical challenges faced in human skeletal reconstruction, where materials must withstand dynamic stresses to prevent graft failure^[17]^. Moreover, defect location introduces variability. Inclusion of sutures (e.g., sagittal or coronal sutures) significantly enhances healing due to residual osteoprogenitor cells in suture mesenchyme^[18]^, yet many studies omit this detail or inconsistently report defect placement relative to anatomical landmarks^[17,19,20]^. For example, defects overlapping the sagittal suture may heal faster than those in suture-free regions, skewing outcomes and reducing reproducibility^[1,18,20]^.

A critical, but often overlooked limitation of the rat CDMs is that they heal exclusively through intramembranous ossification, whereas most clinically significant long bone defects in humans undergo endochondral ossification. In intramembranous healing, as seen in the calvarium, mesenchymal stem cells (MSCs) directly differentiate into osteoblasts to form new bone without a cartilage intermediate^[21,22]^. In contrast, long bone repair involves a more complex process where a cartilage template is first formed and then gradually replaced by endochondral ossification^[21,23]^. As a result, materials that appear highly effective in CDMs may not facilitate the crucial stages of chondrogenesis, cartilage resorption, and vascular invasion required for long bone healing. This gap is further compounded by the lack of mechanical loading in CDMs, which fails to replicate the biomechanical challenges encountered in load-bearing bones^[1,17]^. Therefore, while RCDs are valuable for screening osteoconductive properties, relying solely on them risks overlooking essential aspects of bone regeneration needed for successful clinical translation, particularly for therapies intended for long bone reconstruction.

Current biomaterial and biologic delivery strategies face significant limitations that impact translational calvarial regeneration outcomes. High-dose BMP-2 protein therapies, while effective in stimulating bone formation, are associated with notable risks such as edema, heterotopic ossification, and ectopic bone formation^[24,25]^. These complications arise because supraphysiological BMP-2 doses are often required for robust bone healing, but such concentrations increase the incidence of adverse effects without necessarily improving regeneration rates^[24,26]^. This is particularly relevant in small animal models like RCD, where the local tissue environment and defect size may exacerbate the risk of off-target bone formation and inflammation. Another critical limitation is the mismatch between scaffold degradation rates and the timeline of new bone formation. For example, silk fibroin scaffolds, despite their excellent biocompatibility and mechanical properties, often persist *in vivo* for extended periods, sometimes beyond 18 weeks, without complete resorption^[27,28]^. In RCDs, this slow degradation can impede the natural remodeling process, as residual scaffold material may physically obstruct new bone ingrowth or alter the local microenvironment, thus hindering full defect closure^[28]^. While modifications to scaffold composition and structure can enhance osteogenic differentiation and cellular response, the challenge remains to tailor degradation kinetics precisely to the rate of bone regeneration in specific defect models.

## Translational strategies and future directions

To advance the translational relevance and reproducibility of RCD studies, several actionable refinements are proposed. Establishing standardized defect parameters, including consensus on CSD dimensions, anatomical location, and animal age, is essential. For example, while an 8 mm defect is widely accepted as CSD in rats, evidence suggests that age significantly influences healing capacity and CSD determination; thus, incorporating aged rat models could better mimic clinical scenarios such as osteoporosis or diabetes^[9,10,17]^. Standardization will facilitate more reliable comparisons across studies and improve the predictive value of preclinical findings^[9,17]^. Mandating full compliance with ARRIVE 2.0 guidelines represents a critical step toward standardizing experimental reporting in RCD research^[29]^. The ARRIVE Essential 10 explicitly requires species-specific documentation of animal strain, sex, age, and perioperative conditions to mitigate variability arising from uncontrolled biological and methodological confounders^[29]^. The ARRIVE 2.0 framework addresses these variables through structured checklists that enforce transparent reporting of housing conditions (e.g., ambient temperature and light cycles), surgical protocols, and postoperative care, all of which influence skeletal repair dynamics^[29]^.

The design of advanced biomaterials should focus on developing “smart” scaffolds with tunable degradation rates, such as biphasic microspheres, to synchronize scaffold resorption with the natural stages of bone formation^[9]^. Combination strategies that integrate human umbilical cord MSCs and low-dose biologics may further enhance regeneration while minimizing adverse effects commonly seen with high-dose protein therapies^[30]^. Incorporating mechanistic and long-term studies into CDMs is crucial. The addition of mechanical stimulation systems can provide insights into the role of biomechanical cues in bone healing, while extending observation periods to at least 24 weeks allows for the assessment of late-stage complications, including fibrosis and resorption, which are often overlooked in shorter studies^[31]^.

To bridge the translational gap, prioritizing large animal studies, particularly in rabbits, minipigs, porcine, or non-human primate models, is recommended. These models more closely replicate human cranial biomechanics and the time course of defect healing, thereby providing a more accurate assessment of the clinical potential of new therapies before human trials^[30,32,33]^. Besides, pairing calvarial studies with translational testing in porcine femoral condyle or tibial defect models, which naturally heal via endochondral ossification. This hierarchical and systematic approach ensures that biomaterials validated in rodents are also assessed under clinically relevant healing mechanisms before human trials, thereby ensuring clinically relevant translation while accounting for interspecies regenerative variances^[34]^. Recent studies, however, have shown that it is possible to promote endochondral ossification in RCD by using scaffold-free stem cell constructs or copolymer scaffolds loaded with both chondrogenic and osteogenic factors, which induce cartilage formation followed by bone remodeling^[35,36]^. These findings suggest that refining the CDM to incorporate endochondral healing mechanisms, for example, through the use of specific cell types or growth factor combinations, could bridge the gap between preclinical testing and clinical translation, especially for therapies intended for long bone reconstruction^[35,37]^.

To address the biological constraints inherent in traditional rodent calvarial models, emerging vascularization strategies offer transformative potential for redefining these experimental platforms. Three-dimensional bioprinted microvasculature presents a promising approach for creating hierarchical vascular networks that can support enhanced tissue regeneration^[38]^. Additionally, arteriovenous loop integration provides immediate blood supply, making it particularly suitable for vascularizing larger tissue-engineered constructs^[39]^. Concurrent modulation of the immune microenvironment through targeted macrophage polarization toward anti-inflammatory M2 phenotypes and strategic deployment of cytokine-guided therapies can further optimize the regenerative cascade^[40,41]^. These integrated approaches collectively acknowledge the fundamental limitations of mechanical loading deficiencies and restricted healing mechanisms in current calvarial models.

## Conclusions

The CDM remains indispensable for probing bone regeneration mechanisms, yet its translational value hinges on addressing methodological inconsistencies and biological disparities. By embracing standardized protocols, advanced biomaterials, and clinically relevant testing environments, researchers can transform these models into reliable predictors of therapeutic success, ultimately accelerating the delivery of innovative craniofacial reconstructive solutions. Collectively, these refinements will strengthen the predictive power and clinical relevance of calvarial defect research.

## Data Availability

Not applicable.
